# Burden and Management of Multi-Drug Resistant Organism Infections in Solid Organ Transplant Recipients Across the World: A Narrative Review

**DOI:** 10.3389/ti.2024.12469

**Published:** 2024-06-17

**Authors:** Maristela Pinheiro Freire, Stephanie Pouch, Abi Manesh, Maddalena Giannella

**Affiliations:** ^1^ Department of Infectious Diseases, Hospital das Clínicas, University of Sao Paulo School of Medicine, Sao Paulo, Brazil; ^2^ Transplant Infectious Diseases, Emory University School of Medicine, Atlanta, GA, United States; ^3^ Department of Infectious Diseases, Christian Medical College, Vellore, India; ^4^ Department of Medical and Surgical Sciences, University of Bologna, Bologna, Italy; ^5^ Infectious Diseases Unit, IRCCS Azienda Ospedaliero-Universitaria di Bologna, Bologna, Italy

**Keywords:** solid organ transplant, multidrug-resistant Gram-negative bacteria, epidemiology, clinical impact, diagnosis and treatment, new anti-infective agents

## Abstract

Solid organ transplant (SOT) recipients are particularly susceptible to infections caused by multidrug-resistant organisms (MDRO) and are often the first to be affected by an emerging resistant pathogen. Unfortunately, their prevalence and impact on morbidity and mortality according to the type of graft is not systematically reported from high-as well as from low and middle-income countries (HIC and LMIC). Thus, epidemiology on MDRO in SOT recipients could be subjected to reporting bias. In addition, screening practices and diagnostic resources may vary between countries, as well as the availability of new drugs. In this review, we aimed to depict the burden of main Gram-negative MDRO in SOT patients across HIC and LMIC and to provide an overview of current diagnostic and therapeutic resources.

## Introduction

Solid organ transplant (SOT) recipients are at high risk for acquiring colonization and/or infection with multi-drug resistant organisms (MDRO) with associated high morbidity and mortality rates [1–3].

In the last 10 years, Enterobacterales, *P. aeruginosa,* and *Acinetobacter baumannii* have emerged as critical threats due to a progressive widespread pattern of resistance, impacting patient survival, mainly among vulnerable populations [4]. The present review will focus on these pathogens.

The objective is to provide an overview of the epidemiology of these MDROs in SOT recipients in different regions of the world. Diagnostic and treatment strategies will be also reviewed considering differences in the access to new diagnostic tools and new antibiotics across high- and low-medium-income countries.

## Methods

We conducted a narrative review by a computer-based PubMed search using as keywords “Solid Organ Transplantation,” “multidrug resistance,” “extended-spectrum β-lactamase producing” or “extended-spectrum cephalosporin resistance,” “carbapenem resistance” or “carbapenemase-producing,” “difficult to treat resistant *P. aeruginosa,”* “carbapenem-resistant *A. baumannii”* to identify published all-language literature between June 2013 and June 2023. A pre-established chart was used to extract epidemiological data. MDRO was defined according to Magiorakos criteria and new DTR concept [5,6]. HIC and LMIC were defined according to world bank classification [7]. To estimate MDRO prevalence in SOT recipients across countries, we included studies reporting the number of infections by each specific MDRO, as well as the number of transplanted patients during the same period. Studies that only reported colonization or laboratory-based descriptions without clinical data were excluded.

## Results

### Epidemiology of MDRO Infections After SOT

**FIGURE 1:**
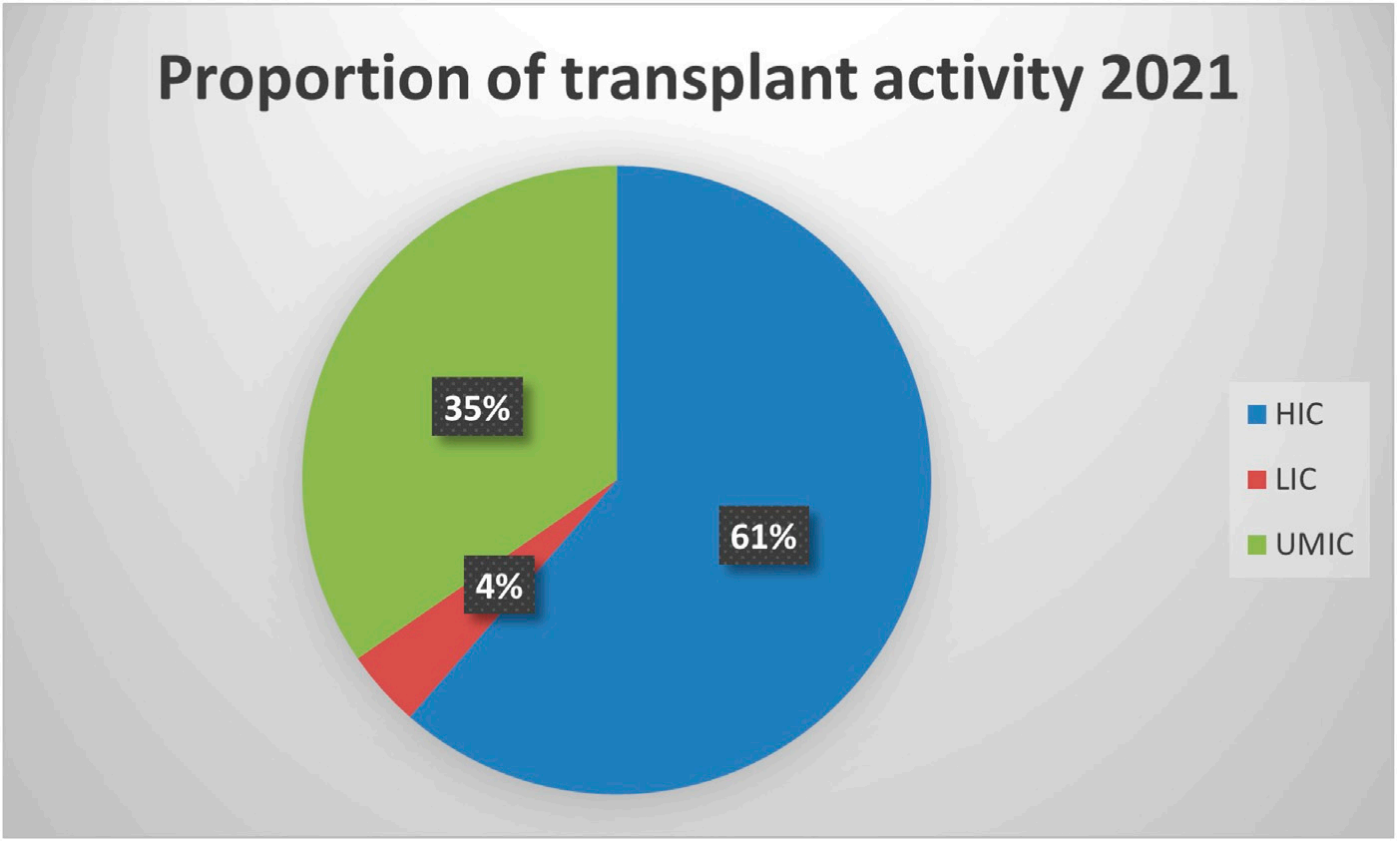
Proportion of transplant activity in high-, lower- and upper middle-income countries.

**FIGURE 2:**
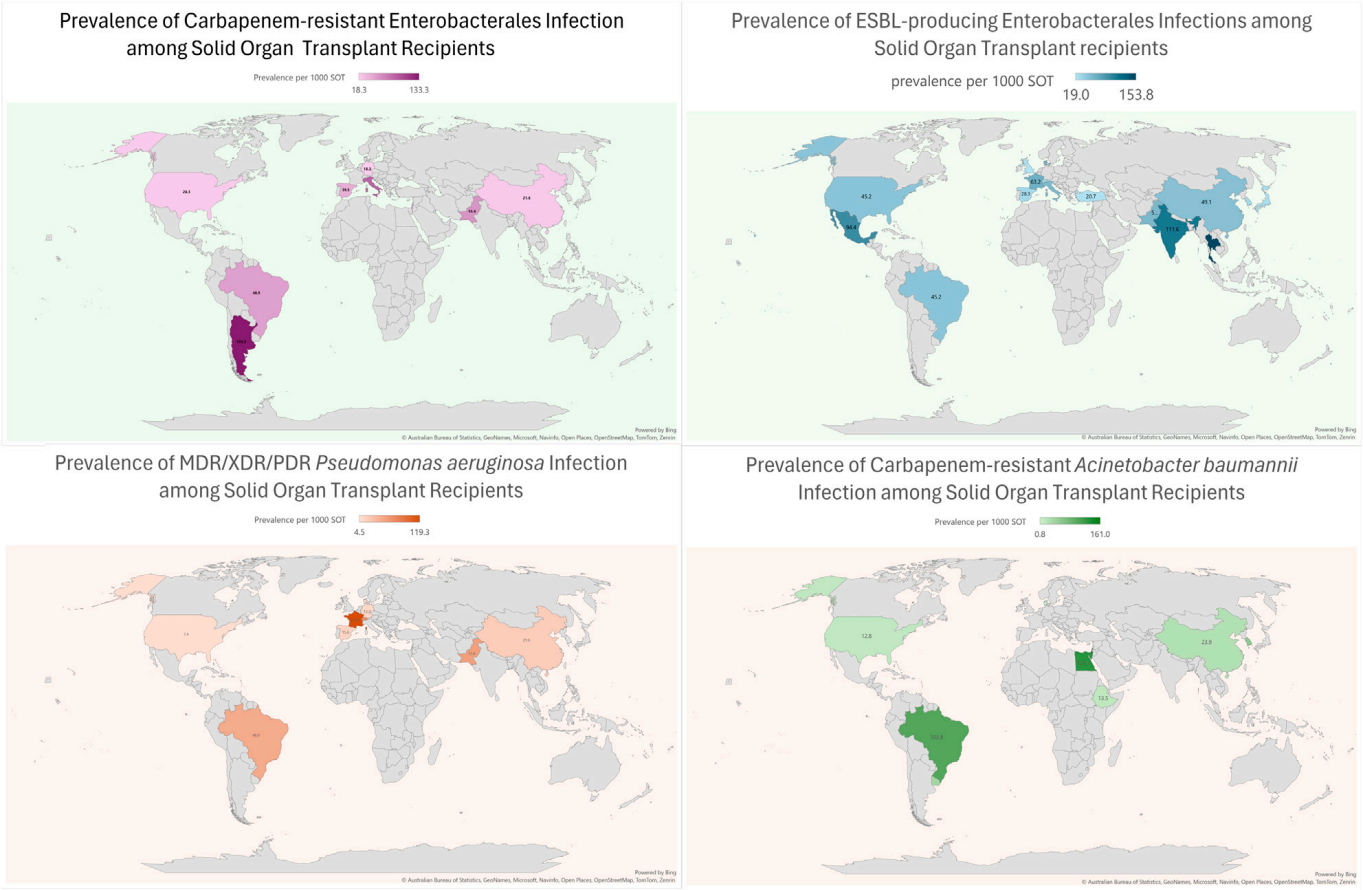
Prevalence of Multidrug resistant Gram-negative bacterial infections in SOT recipients across the world.

Compared to high-income countries (HICs), data on MDRO infections among SOT patients is relatively scarce in low and middle-income countries (LMICs). In these regions, the number of transplants per million people is lower when compared to Western Europe and the US. However, in absolute terms, 39% of all transplants are performed in these countries (see Figure 1) [8]. Significant discrepancies in donor referral and transplantation exist between HICs and LMICs. In the latter, the proportion of living-donor transplants is higher, especially in Asia [9]. Moreover, the rates of MDRO infections among SOT recipients are highly influenced by the local epidemiology. For instance, Brazil, Turkey, India, China, and Argentina are described as countries with the highest prevalence of CRAB infection [10] Moreover, India and China have a high prevalence of ESCR-E and CRE, mainly NDM-producing [10,11]. Thus, it is expected that LMIC bear a high burden of these diseases, which are likely underreported due to deficiencies in diagnosis, lack of microbiology laboratory infrastructure, and limited resources to make post-transplant infection rates public. Finally, there is a lack of representativity from countries in the Middle East and Africa. Taking into account these considerations, an overview of the worldwide prevalence of infection by most common MDROs per 1,000 transplant-recipients is shown in Figure 2.

#### ESBL-Producing Enterobacterales

ESBL-E infection is the most commonly reported MDR Gram-negative infection, with a prevalence ranging from 3% to 11% and an aggregate rate of 7% among all bacterial infections in all types of SOT; however, in KT recipients the prevalence of ESBL-E, mainly in urinary tract infections (UTIs) may be >30% in high endemic centers [12–33] (Table 1). Data from the Swiss Transplant Cohort showed that, ESBL production was observed in 11.4% Enterobacterales isolated from 1072 SOT recipients [70]. Enterobacterales infections occurred at a median of 69 days after transplant, interestingly patients were predominantly outpatients. Higher prevalence of ESBL-E has been reported in studies analyzing SOT recipients with BSI and UTI [36,37,71]. In a study assessing the epidemiology of UTI in a cohort of 4388 SOT recipients in Spain, the prevalence of ESBL in *E. coli* was 26% [38].

**TABLE 1:** Prevalence of MDRO infections in SOT recipients reported in studies from low- and medium-income and high-income countries.

	Low and medium-income countries				High-income countries			
Type of resistance	All infections	BSI	UTI^a^	LRTI	All infections	BSI	UTI	LRTI
ESBL	7.0% (4.4%–11.2%)	3.4% (0.9%–11.7%)	14.4% (5.6%–21.6%)	NA	5.5% (2.2%–13.6%)	12.8% (7%–40%)	5% (1%–6%)	NA
CRE	4.0% (0.9%–15.7%)	2.0% (0.9%–7.8%)	2.8% (0.8%–7.7%)	1.3% (1%–2.1%)	6% (1.9%–10.3%)	8%	NA	NA
DTR-Pa	1.4% (0.8%–3.9%)	3.1% (1.5%–8.0%)	1.1% (0.8%–1.5%)	3.2%	7%	10%	NA	9% (3%–15%)
CR-Ab	4.1% (0.8%–28.6%)	1.4% (1.1%–28.6%)	NA	5.8% (3.8%–9.7%)	1%–6%	NA	NA	NA

^a^ Rates of UTI, are mainly obtained from studies including kidney transplant recipients.

References: ESBL LMIC [13,16–21,34,35]; ESBL HIC [22–33,36–41]; CRE LMIC [14,16,20,42–47]; CRE HIC [39,48–53]; DTR-Pa LMsIC [14,16,46,54–58]; DTR-Pa HIC [2,22,49,52,59–61]; CR-Ab LMIC [15,16,20,46,57,62–65]; CR-Ab HIC [22,66–69].

Two large studies have investigated molecular characteristics of MDR-E isolated in SOT recipients, from Spain and US each. In the Spanish study, 541 MDR-E isolates were collected. The main microorganisms were *E. coli* (46.2%), *K. pneumoniae* (35.3%), *E. cloacae* (6.5%) and *C. freundii* (6.3%). Overall, 78.0% of strains harbored ESBL genes, CTX-M-group-1 being the most prevalent (53.3%) followed by CTX-M-group-9 in 15.4%. Among ESBL-producers, 2.1% of *E. coli*, 47.3% of *K. pneumoniae* and 11.1% of *E. cloacae* harbored a carbapenemase gene. Hyperproduction of chromosomal-AmpC was detected in *E. cloacae* (57.7%), *C. freundii* (82.6%) and other MDR-E species (39.1%) [72]. In the US study on 88 transplant recipients, 20% of patients were colonized with MDR-E (ESCR-E only *n* = 23; CRE only *n* = 12; both *n* = 5), 52% of ESCR-E carried blaCTX-M. Post-transplant MDR-E infection rate was 10%, the attack rate was higher following CRE than ESCR-E colonization (53% vs. 21%, *p* = .05) [39].

Main risk factors for ESBL-E infections after SOT are reported in Table 2 [73]. In this regard, the role of targeted perioperative antibiotic prophylaxis (T-PAP) is still an open issue [74]. Two studies, from France and Thailand each, showed a reduction of ESBL-E infections in patients receiving T-PAP after OLT and KT, respectively [13,40]. However, both of them showed several limitations including observational design, heterogeneity in drugs used in the OLT study, and consideration of asymptomatic bacteriuria as an endpoint in the KT cohort. Furthermore, it should be remarked that carbapenem exposure is the main driver for carbapenem resistant infections.

**TABLE 2:** Risk factors for MDRO infection and for mortality.

Type of resistance	Risk factors for infection	Risk factors for mortality
ESBL-E [13,34,40,70,71,73]	*General Characteristics* - Female gender - Kidney Transplant - MELD score >25 *Colonization status* - ESBL-Enterobacterales carriage in the prior 1 year *Pre-SOT antibiotic exposure/prophylaxis* - Pre-operative prophylaxis for spontaneous bacterial peritonitis - Carbapenems prophylaxis - Exposure to third-generation cephalosporin, TMP/SMX or echinocandins in the prior 6 months *Post-SOT condition* - Acute rejection in prior 3 months - Reoperation - Corticosteroid containing immunosuppressive regimen	*Severity of patient and/or condition* - Pitt bacteremia score - Mechanical ventilation at the time of infection diagnosis
CRE [42,43,48,74–83]	*General Characteristics* - Male gender - Older age - Time of hospitalization - Lung transplant - Liver transplant - Multiple infected organisms or sites - Previous infections - Dialysis - MELD score >32 - Median lymphocytes count under 700 cell/mm3 *Colonization status* - Pre/post-transplant CRE carriage - Multisite colonization - Colonization by more than one species of CRE *Pre-SOT antibiotic exposure* - Carbapenem use (OR 2.53, OR 2.80) *Post-SOT condition* - Combined transplant - Prolonged mechanical ventilation - Possible donor-derived infection - Delayed kidney function/Ureteral stent - CMV infection - Re-transplantation - Rejection - Mycophenolate use	*General Characteristics* - older age - CMV disease - Lymphocytes ≤600 U/mm^3^ - Pitt bacteremia score - Graft failure *Severity of patient and/or condition* - Septic shock - High SOFA score - Multiple infected organisms or sites - Genitourinary source - No source control - INCREMENT-CPE mortality score ≥8 *Antibiotic exposure* - Appropriate empiric therapy (protective) - Polymyxin exposure in the prior 6 months
DTR-Pa [54,59–61,84,85]	*General Characteristics* - *Hospital stay > 10* *days* - *Lower median lymphocyte counts* - *Central venous catheter* - *Urinary catheter* - *Prior transplantation* - *ICU admission in previous year* - *Septic shock* *Pre-SOT antibiotic exposure* - *Prior carbapenem use* - *Prior ciprofloxacin use* *Post-SOT condition* - *re-transplantation* - *urological surgical procedure after Kidney transplant*	*Severity of patient and/or condition* - Bacteremia - creatinine >1,5 - onset of BSI while in ICU
CR-Ab [20,44,62,86–89]	*General Characteristics* - *Liver Transplant performed because of fulminant hepatitis* - *high preoperative serum levels of BUN* - *pre-operative hypoalbuminemia* *Post-SOT condition* - *Fungal culture positivity after SOT* - *long duration of surgery* - *tracheal intubation twice* - *longer cold ischemia time* - *post-Liver transplant need for dialysis*	*Severity of patient and/or condition* - *Platelet count < 50,000/mm3* - *Mechanical ventilation at the onset of CRAb* - *ICU-acquired infection* *Antibiotic exposure* - *Inappropriate empiric therapy* - *Colistin-carbapenem regimens*

ESBL-E infections are associated with increased length of stay, mainly in case of initial inappropriate therapy [34,35]. In addition, high recurrence rates have been reported ranging from 25% for BSI to 79% for UTI, mainly in KT recipients [34,90]. Factors associated with relapse were inappropriate empirical therapy, advanced age, and persistent bacteriuria [41,70].

#### Carbapenem-Resistant Enterobacterales

The prevalence of CRE infection after SOT varies according to the type of organ, being higher among liver (2%–10%) and lung (5%–7%) transplant recipients [91]. These rates seem to be a little higher in HIC than in LMIC (see Table 1) [42–45,49–52]. The rate of CRE infection is on average 30% among CRE carriers [53]. Usually, CRE infection occurs in the first 4–8 weeks after transplant, earlier infections (within 2 weeks) are observed in pre-transplant carriers and/or in donor derived infections (DDI) [48,53,92]. Notably, incidence of DDI due to CRE is high in China, one study focused on KT patients reported that possible DDI increased the risk of CRE infection by more than six times [75]. Authors reported varying prevalence rates of CRE among donor or preservation fluid cultures, ranging from 1.6% to 19.2% [93–95].

CRE infection after SOT often presents as severe infection with BSI and/or lung involvement [76,91].

Carbapenemases show significant geographical variation—*K. pneumoniae* carbapenemases (KPC) remain the commonest in United States, the metallo-beta-lactamases (MBLs) are most common in the countries of South and Southeast Asia and OXA-48-type carbapenemases in the Middle East, Mediterranean and northern African countries [96–98]. In the two studies assessing molecular characteristics of MDR-E isolated from SOT recipients, the main mechanisms of carbapenem resistance were OXA-48 in Spain accounting for 78% of the isolates, and KPC in US detected in 72% of CRE [39,72]. These mechanisms were mostly detected in *K. pneumoniae* isolates. Few studies in LMICs investigated this issue. The proportion of strains with carbapenemase-producing is reported to be 46%–84% among OLT recipients and 83% among KT recipients. KPC-producing CRE appears to be the most frequent. The second most common carbapenemase is NDM, which corresponded to 28% in an OLT cohort in China and 2% in a KT cohort in Brazil. Despite, CRE post-transplant infection rates are high in India, details about the proportion of NDM and KPC are not available [99]. Other carbapenemases, such as IMP, are less frequent and often associated with outbreaks [77,78,100,101].

Risk factors for CRE infection have been usually investigated in specific organ transplant settings and most commonly in OLT recipients (see Table 2) [48,75,79,80]. Carriage, either acquired before or after transplant, and peri-surgical complications have been associated with highest risk of developing CRE infection [48,77]. For pre-transplant carriage, shorter the time of detection before SOT, higher is the risk of infection after SOT [81]. For post-transplant carriage, it is worth mentioning that this occurs 2-3 times as more frequently than pre-transplant carriage, thus in high endemic areas it could be considered to repeat the screening for rectal carriage, which is usually done before or at transplant time, also during the post-transplant period during ICU or hospital stay. Conversely, the role of T-PAP for CRE is under debate [74,82].

Rates of mortality and graft failure in patients developing CRE infection after SOT are as high as on average 40% and 20%, respectively. After adjusting for confounding variables CRE infection was found as a significant predictor of poor outcome [83].

#### Difficult-To-Treat Resistant P*seudomonas aeruginosa*

Assessing the burden of difficult-to-treat resistant (DTR) *P. aeruginosa* (DTR-Pa) in SOT recipients is difficult for several reasons including: i) different drug resistance definitions used across centers and study periods; ii) analysis of respiratory isolates generally available only for LuT recipients while for other types of transplant most data come from studies on BSI; iii) cumulative data on drug resistance provided including also other pathogens; and iv) lack of large multicenter studies.

With this premise, DTR-Pa appears to be the MDRO with the lowest prevalence among SOT in LMIC, described from 0.8% to 3.9% in KT and OLT recipients (Table 1) [54–58]. In HIC, Pa generally ranked first among pathogens isolated from LuT recipients, with rates of MDR ranging from 7% to 50% [59,60,84]. In a single-center Spanish study, including 318 consecutive episodes of BSI in a cohort of non-lung SOT recipients, 44 (15%) BSI were caused by Pa with 31 (63%) strains classified as XDR [61]. The most frequent source was UTI, and the median time from transplantation to BSI was shorter for XDR episodes (66 vs. 278 days). Independent risk factors for XDR Pa BSI were prior transplantation, nosocomial acquisition and septic shock [85]. Only colistin and amikacin maintained activity against XDR strains. Compared to patients with susceptible-Pa BSI, those with XDR-Pa BSI received more frequently inappropriate empirical treatment (58% vs. 22%), and had higher 7-day (20.7% vs. 8.5%) and 30-day (38% vs. 16%) mortality rates.

Few data are available about the mechanisms underlying DTR and CR phenotypes in Pa. In a recent study including CR-Pa from 972 individuals (USA *n* = 527, China *n* = 171, south and central America *n* = 127, Middle East *n* = 91, Australia and Singapore *n* = 56), almost a quarter of strains were shown to produce a carbapenemase, mostly consisting of KPC-2 (49%) or VIM-2 (36%), with a prevalence varying across south and central America 69%, Australia and Singapore 57%, China 32%, Middle East 30%, US 2% [4]. In a study on 163 clinical *P. aeruginosa* isolates in adult cystic fibrosis and LuT in Australia, 32 (19.6%) were XDR, 82% of strains were susceptible to ceftolozane/tazobactam [102].

Mortality risk associated with DTR/XDR or CR Pa infection after SOT seems to be higher in patients with septic shock and/or multiorgan failure and ICU stay [61].

#### Carbapenem-Resistant *Acinetobacter baumannii*

The overall rate of CRAb infection among SOT recipients varies from 1% to 6% in HIC [66–69]. In a study conducted in US on 248 patients with *A. baumannii* infection, CRAb rates were higher among SOT compared to non-SOT patients (43% vs. 14%) [103].

CRAb prevalence after SOT in China and Brazil can reach 29% [10,16,62,63,86]. A systematic review focused on uro-pathogens among KT recipients highlighted *A. baumannii* as the third most frequently encountered Gram-negative bacteria, displaying a prevalence rate of 8% in the Middle-East [104] Additionally, 4%–10% of OLT recipients have pneumonia attributed to CRAb in China, Brazil, Egypt and Uruguay [64,65,87,88] A Chinese study involving 107 LuT recipients found that CRAb was the predominant MDRO infection agent, accounting for 35% of Gram-negative MDRO [63]. Thus local epidemiology is pivotal in planning screening for CRAb before and after SOT.

*A. baumannii* is intrinsically resistant to a wide range of antibiotic classes, caused by simultaneous mechanisms of resistance [105]. Among these, decreased outer-membrane porins, constitutional expression of efflux pumps, intrinsic harboring of β-lactamases and plasmidial carbapenemase, has been widely described. Among carbapenemases, OXA-23-like are the most common. However, CRAb isolates harboring MBL, such as blaNDM-1 genes, has been associated with increased mortality rates in a study conducted from Pakistan [106–108]. Resistance to polymyxin is infrequent and appears to be linked to outbreaks [46,62,86,109].

Data about risk factors for CRAb infections were exclusively reported for OLT recipients from LMICs [87,88]. (Table 2) CRAb infection mortality rates are the highest among SOT MDRO infections and often exceed 40% (ranging from 20% to 47%) [62,86,87,89,110].

### Diagnosis of MDRO Infections After SOT

Timely diagnosis of MDRO infections in SOT recipients is critically important to patient and allograft survival. Advanced diagnostic methodologies may aid in shortening the time to narrowest appropriate antibiotic administration; however, data on their optimal use and interpretation in this specific population are limited [111]. In addition, the availability of rapid diagnostics may vary by location.

In a survey among American Society Transplant (AST) members, 19 respondents indicated frequently ordering multiplexed molecular assays (82%) and antimicrobial susceptibility to new antibiotics (76%), and >80% of respondents reported to change treatment according to the results of such tests [112]. However, data from other countries are missing.

Preliminary data on the use of a multiplex PCR panel in 29 transplant recipients with 45 bloodstream infections remarked the possibility of off-target pathogens [113]. Indeed, a consensus conference to define the utility of these new diagnostics in SOT concluded that prospective multicenter studies are needed to investigate their performance and reproducibility compared to reference standards, the optimal timing of testing to predict and/or diagnose disease, the impact on clinical outcomes, and the cost-effectiveness also for point-of care applications [112].

#### ESBL-Producing Enterobacterales

Molecular detection of ESBL genes may aid in decreasing the time to diagnosis and initiation of targeted antimicrobials in SOT recipients. Several systems capable of detecting ESBL-producing Enterobacterales in lower respiratory tract specimens and blood are commercially available; however, not all genes responsible for ESBL production, including *bla*_*TEM*_ and *bla*_*SHV*_ are included on all panels. Moreover, assays used for rapid genotypic resistance detection display reduced accuracy in polymicrobial infections [111,112]. Rapid phenotypic antimicrobial susceptibility testing has also been demonstrated to reduce the time to optimal therapy among bacteremic non-transplant patients [111,114].Current recommendations underscore the need for conventional antimicrobial susceptibility testing to verify results of rapid genotypic and phenotypic testing when there is concern for a highly resistant phenotype and for polymicrobial infection [1,111].

#### Carbapenem-Resistant Enterobacterales

Several rapid diagnostic tests for carbapenem resistance are commercially available and include real‐time polymerase chain reaction and nucleic acid tests such as the Xpert^®^ Carba‐R (Cepheid), Verigene^®^ BC‐GN (Luminex), and BioFire^®^ FilmArray^®^ Blood Culture Identification 2 Panel, which test for *bla*_*KPC*_, *bla*_*NDM*_, *bla*_*OXA*_, *bla*_*VIM*_, and *bla*_*IMP*_ gene sequences [115,116].However, this assays display reduced accuracy in polymicrobial infections [111,112]. Other methods for rapid diagnosis of CRE include chromogenic assays as RAPIDEC^®^ CARBA NP (bioMérieux) and Rapid CARB Blue (Rosco Diagnostics) and matrix‐assisted laser desorption ionization‐time-of-flight mass spectroscopy (MALDI‐TOF MS) [117–119]. While rapid diagnostic assays for the detection of carbapenem resistance may reduce the time to effective antimicrobial therapy, current guidelines and expert consensus recommendations recommend conventional antimicrobial susceptibility testing to confirm the diagnosis of a CRE infection [1,111].

According to local availability, antimicrobial susceptibility to old and new agents is advisable not only on the clinical isolate but also on the colonizing strain in order to start promptly an appropriate treatment upon the onset of infection symptoms/signs.

#### Difficult-To-Treat Resistant *Pseudomonas aeruginosa*

Difficult-to-treat resistance has been defined as *P. aeruginosa* which exhibits non-susceptibility to aztreonam, piperacillin-tazobactam, ciprofloxacin, levofloxacin, ceftazidime, cefepime, meropenem, imipenem-cilastatin [120].

Rapid diagnostic tests for the identification of *P. aeruginosa* are commercially available and include nucleic acid tests, MALDI-TOF MS, and peptide nucleic acid fluorescent *in situ* hybridization (PNA FISH; AdvanDx) [121]. However, given that DTR-Pa evolves due to multiple resistance mechanisms, current guidelines recommend against rapid diagnostic testing to guide empiric treatment [1].

#### Carbapenem-Resistant *Acinetobacter baumannii*

In low-prevalence areas, use of rapid diagnostic and phenotypic tests for the detection of CRAB has posed clinical challenges. A recent study comparing the NG-Test CARBA 5 (NG-Biotech) version2 with the Xpert-Carba-R assay, modified carbapenem inactivation method (mCIM), and the CIMTris assay with whole-genome sequencing as the reference standard demonstrated that the NG-Test CARBA 5 and Xpert Carba-R had an overall percentage agreement of 6.2%, noting OXA-type carbapenemases are not included, and the CIMTris had an overall percentage agreement of 99%. In addition, approximately 96% of isolates incorrectly tested positive for IMP on NG-Test CARBA 5 [122]. Supplementary studies are being undertaken to identify opportunities for rapid diagnostics for CR-Ab infections [123,124].

### Management of MDROs in SOT patients

**FIGURE 3:**
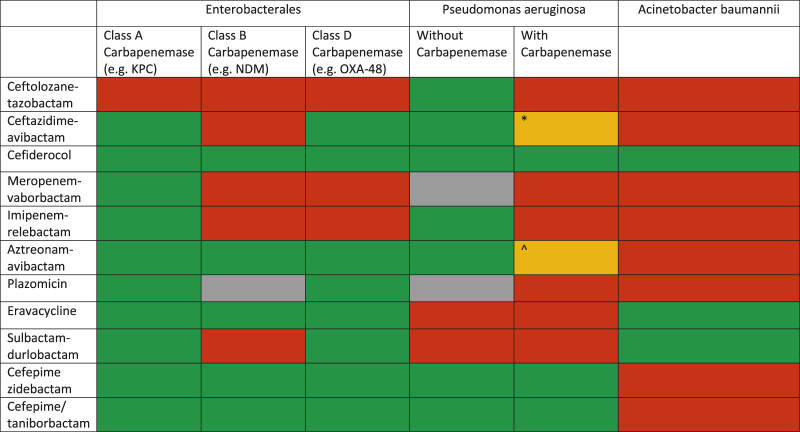
Spectrum of various novel agents active against carbapenem-resistant Gram-negative organisms (modified from: European Respiratory Review 2022 31: 220119) *This drug may retain activity against serine-type carbapenemases (e.g., GES) but are inactivated by metallobetalactamases ^This combination has been shown to be active *in vitro* against some MBL producing *P. aeruginosa* strains.

**FIGURE 4:**
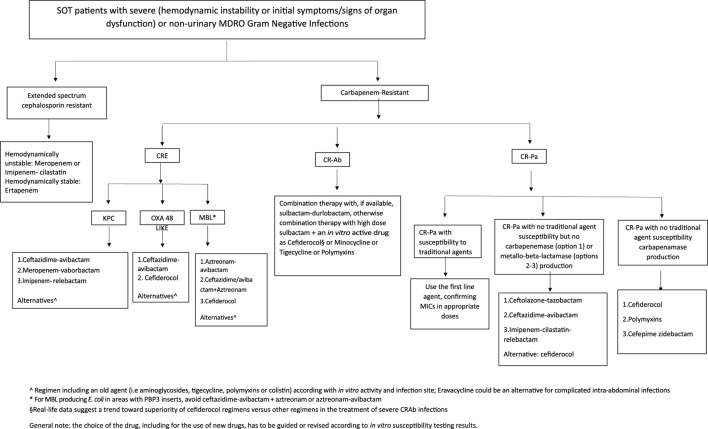
Treatment flowchart.

The management of MDRO infections in SOT patients is not different from that recommended in other patients in view of choice agent/regimen and treatment duration. The outsized burden of AMR in the LMICs is further complicated by non-availability of recently approved antibacterial agents. For example, in the South Asian region, where carbapenem-resistant infections are very common, cefiderocol, sulbactam-durlobactam, meropenem-vaborbactam, imipenem-relebactam, eravacycline and plazomicin are not yet available. The treatment for severe infections, with bacteraemia as a prototype, is discussed here. Overall, spectrum of activity of various antimicrobial agents is shown in Figure 3. Selection of agents should be based on *in vitro* activity and local availability. An algorithm for treatment approaches is proposed in Figure 4.

#### Third-Generation Cephalosporin-Resistant Enterobacterales

Carbapenems are considered the drug of choice for the management of severe ESBL-E infections in SOT patients [120,125]. The MERINO trial compared piperacillin-tazobactam versus meropenem for the management of ceftriaxone-resistant *E. coli* and *Klebsiella* spp. bacteremia. Thirty-day mortality, was higher in the piperacillin-tazobactam group (12% vs. 4%; absolute risk difference 9%), failing to meet the non-inferiority margin [126]. The carbapenem superiority appears to be related to elevated piperacillin-tazobactam MICs with co-occurrence of narrow spectrum oxacillinases [127]. Ertapenem is generally deferred as an upfront therapy in critically ill patients [128]. Carbapenems including ertapenem, fluoroquinolones, TMP-SMX and aminoglycosides are options for stable patients with pyelonephritis and other UTI. Switch to oral regimens can be considered once clinical stability is achieved and susceptible oral agents with good intestinal absorption are available [125].

*Klebsiella aerogenes*, *Enterobacter cloacae* complex, and *Citrobacter freundii* are commonly associated with higher risk of AmpC-β-lactamase production [129]. Despite its limited ability to induce AmpC-β-lactamases, piperacillin-tazobactam is considered inferior for treatment due to the risk of hydrolysis [130]. MERINO 2, a small RCT evaluating piperacillin-tazobactam versus meropenem in bacteremia with presumed AmpC-producing organisms showed no difference between the two agents in clinical failure and mortality [131]. However, some observational data point to poorer outcomes with piperacillin-tazobactam [132–134]. Cefepime minimally induces AmpC β-lactamases and is relatively stable to AmpC hydrolysis. Some observational studies show higher mortality with cefepime MICs 4–8 μg/mL (susceptible dose-dependent range), probably correlating with co-production of ESBLs [135]. Carbapenems are stable against AmpC β-lactamases and are the drugs of choice for severe infections and/or upon isolates with MICs ≥4 μg/mL for cefepime [120].

Studies addressing the role of intestinal decolonization for SOT recipients colonized with ESCR-E are limited. One case-control study described the successful use of a 5-day course of norfloxacin in reducing the burden of ESBL-E in stool samples obtained from OLT recipients during an outbreak in a transplant unit [136]. However, other studies have described the development of colistin- and tobramycin-resistant *K. pneumoniae* after attempted decolonization with orally administered colistin [137,138]. Given the risk of selecting resistant organisms, this approach is not recommended [139].

#### Carbapenem-Resistant Enterobacterales

Once the CRE is confirmed, carbapenamase testing and antimicrobial susceptibility for all available agents are recommended. For KPC-producing CRE isolates, ceftazidime-avibactam, meropenem-vaborbactam, and imipenem-relebactam are the first line options of therapy [125]. Cefiderocol can be used provided susceptibility testing is available. For OXA-48 type carbapenemase-producing CRE, ceftazidime-avibactam is the preferred agent of choice. Cefiderocol is an alternative [140,141]. NDM-producing CRE is best treated with a combination of ceftazidime-avibactam and aztreonam. Aztreonam retains activity against MBL but is inactivated by coexistent ESBLs, AmpCs or OXA-48 like enzymes. Avibactam protects the aztreonam from these mechanisms. Cefiderocol is a potential option for treatment of NDM- and other MBL-producers if the isolate is susceptible to this agent. In MBL-producing *E coli,* presence of four-amino acid (YRIN or YRIK) inserts in Penicillin binding protein 3 (PBP3) are common in countries like India and China, reducing the interaction of aztreonam at that site, leading to higher MICs [142,143]. The efficacy of ceftazidime-avibactam plus aztreonam may not be retained in MBL producing *E coli* isolates with PBP3 inserts.

Few studies have assessed the efficacy of the new drugs specifically in SOT recipients. Most data are available for ceftazidime-avibactam as it was first introduced in Europe and US. A multicentre observational study of 210 SOT recipients with BSI due carbapenemase producing *K. pneumoniae*, 149 received active primary therapy with CAZ-AVI (66/149) or best available treatment (BAT) (83/149). Patients treated with CAZ-AVI had higher 14-day (80.7% vs. 60.6%, *p* = 0.011) and 30-day (83.1% vs. 60.6%, *p* = 0.004) clinical success and lower 30-day mortality (13.25% vs. 27.3%, *p* = .053) than those receiving BAT. In the adjusted analysis, CAZ-AVI increased the probability of clinical success; in contrast, it was not independently associated with 30-day mortality. In the CAZ-AVI group, combination therapy was not associated with better outcomes [144].

There is a paucity of data regarding intestinal decolonization of SOT recipients colonized with CRE. A clinical trial on SOT colonized with MDRO failed to show a benefit from decolonization with oral colistin plus neomycin, conversely decolonization was associated with adverse events [145]. Thus, this approach is currently not recommended. The role of fecal microbiota transplantation in restoring intestinal microbial diversity in SOT recipients colonized with MDROs seems promising; however, more data on clinical effectiveness and safety are needed [146].

#### Difficult to Treat Resistant *P. aeruginosa*

Treatment of Pa with carbapenem resistance can be approached in three ways. If a traditional agent like piperacillin-tazobactam, cefepime, ceftazidime or fluoroquinolones remains susceptible with carbapenem resistance, they can be used in optimal doses [147]. This is primarily due to lack of functional OprD which is required for carbapenem entry.

If there is resistance to traditional agents and to carbapenems (e.g., a XDR or DTR strain), it is important to check for carbapenemases [148,149]. If carbapenemase testing is negative, ceftolozane-tazobactam is considered the drug of choice when *in vitro* activity is confirmed. For CR-Pa where resistance is mediated by a non-MBL carbapenemase (e.g., KPC, GES) ceftazidime-avibactam or imipenem-relebactam could be used; cefiderocol is an alternative option.

For CR-Pa isolates with documented MBL production, the therapeutic options are limited. Cefiderocol or polymyxins are generally the only drugs maintaining *in vitro* activity. However, data on clinical efficacy are controversial for cefiderocol, and generally poor for polymyxin/colistin mainly due to toxicity. The combination of ceftazidime-avibactam and aztreonam could be an option although clinical experience is limited [150,151]. Cefepime-zidebactam has been reported as a salvage option in these patients [152,153].

#### Carbapenem Resistant *Acineotacter baumannii*

The therapy of CRAb infections is particularly complex in view of difficulty in differentiating between colonization and invasive infection, especially in the lung, with extremely limited therapeutic options. There is no single antibiotic available as a preferred agent in the management of CRAb infections. One of the recent promising agents is sulbactam-durlobactam. In a phase 3 RCT, 28-day all-cause mortality was 19% in the sulbactam–durlobactam group and 32% in the colistin group, an absolute difference of −13.2%, meeting the non-inferiority criteria. In both groups, combination with imipenem-cilastatin was used. Most guidelines currently recommend sulbactam based therapy and wherever possible in combination with other *in-vitro* active agents [125]. Sulbactam is a competitive betalactamase-inhibitor with independent anti-*Acinetobacter* activity via saturation of PBP1 and PBP3 in high doses [154]. But the susceptible MIC range for sulbactam is not established. Also, changes in the above PBPs can decrease its affinity and result in resistance. Few studies have supported the benefit of ampicillin-sulbactam especially against polymyxins [105]. The options for combination therapy with sulbactam include minocycline, tigecycline, polymyxins and cefiderocol. Colistin is frequently active *in vitro*; however, the unfavourable PK/PD profile of this drug results in low efficacy and high toxicity rates. Two large randomized controlled studies have shown the addition of high-dose meropenem to colistin does not result in clinical benefit [155,156]. Nebulised polymyxins are not currently recommended in view of preferential distribution to the unaffected areas of the lung, absence of benefit in randomized trials and potential for bronchospasm [157–159]. The role of cefiderocol is debated [160,161]. This drug shows high rates of *in vitro* activity and, despite it was associated with higher mortality compared with standard treatment (mostly consisting of colistin-based regimens) in patients with CR-Ab infections in the phase III CREDIBLE-CR trial [161], it has been shown to be more or equally effective than older regimens, with a significantly lower toxicity, in several real-word observational studies [162].

## Conclusion

To conclude, to draw the global burden of MDROs in SOT recipients is difficult due to the lack of standardization in screening and reporting colonization and/or infections with such pathogens; and the access to diagnostic and therapeutic resources could be variable across countries. To improve outcomes associated with MDRO colonization and/or infections in SOT recipients, new rapid advanced diagnostics could be supportive, as well as the prompt availability of phenotypic susceptibility to old and new drugs. Use of these tests should be guided by local epidemiology and patient risk factors, their impact on outcome should be investigated.

## Abbreviation

SOT, solid organ transplantation; MDRO, multi drug resistant organisms; ESBL-E, extended spectrum beta-lactamase producing enterobacterales; ESCR-E, extended-spectrum cephalosporin resistant-enterobacterales; CRE, carbapenem resistant enterobacterales; CPE, carbapenemase producing enterobacterales; KPC, *Klebsiella pneumoniae* carbapenemase; MBL, metallo-beta-lactamase; NDM, New Delhi metallobetalactamase; VIM, verona-integron-metallo beta lactamase; IMP, imipenemase; MDR, multi-drug-resistant; XDR; extensively drug resistant; PDR, pan-drug resistant; DTR, difficult-to treat resistance; CR-Pa, carbapenem resistant *Pseudomonas aeruginosa*; CR-Ab, carbapenem resistant *Acinetobacter* baumannii; HIC, high income countries; LMIC, low and medium income countries; OLT, orthotopic liver transplantation; KT, kidney transplantation; LuT, lung transplantation; HT, heart transplantation; UTI, urinary tract infection; DDI, donor derived infection.
